# Limited matching of the cardiac output response to the peripheral demand of heat stress and exercise

**DOI:** 10.1113/EP092688

**Published:** 2025-03-20

**Authors:** Moritz Lampkemeyer, Jonas Kell, Veit Börß, Tobias Claussen, Fabian Spahiu, Michelle Ottlik, Lars C. Helbig, Craig G. Crandall, Eric J. Stöhr

**Affiliations:** ^1^ COR‐HELIX, Institute of Sport Science Leibniz University Hannover Germany; ^2^ Division of Cardiology, Department of Internal Medicine University of Texas Southwestern Medical Center, Institute for Exercise and Environmental Medicine Dallas Texas USA; ^3^ Division of Cardiology Columbia University Irving Medical Center New York New York USA

**Keywords:** blood flow, blood flow restriction, cardiovascular control, cardiovascular response, exercise, exercise physiology, heat stress

## Abstract

It is widely accepted that cardiac output matches the prevailing peripheral demand in healthy humans. However, it remains unknown whether stroke volume and heart rate are regulated interdependently to arrive at a specific cardiac output. The aim of this study was to determine whether the healthy human heart responds specifically according to the peripheral demands of heat stress and exercise. Eleven healthy humans (women/men *n* = 3/8; age = 26 ± 2 years; body mass = 73 ± 11 kg) underwent leg heat stress and cycling exercise (60 W), with and without blood flow restriction (pressure set at the prevailing mean arterial pressure of the individual). Cardiac output was measured with triplane echocardiography. Additionally, haemodynamics, oxygen consumption, carbon dioxide production and lactate were assessed. Data were analysed using two‐way repeated‐measures ANOVA. Despite stable heat and exercise demands, cardiac output decreased significantly with blood flow restriction in both conditions (Δ−0.87 and −1.03 L min^−1^, 17% and 11%, respectively, *p *= 0.01), owing to a decline in end‐diastolic volume (*p *< 0.0001) and stroke volume (*p *< 0.0001) not sufficiently compensated for by an increase in heart rate (*p *= 0.001). Importantly, these responses were accompanied by an increased rate of skin temperature rise (*p *= 0.04) during heat stress and a significantly greater rise in circulating lactate (*p *< 0.0001) during exercise. The cardiac output response to local heat stress and submaximal exercise does not appear to be entirely specific to the peripheral thermal and energetic requirements. This finding supports the theory that even the healthy heart does not coordinate stroke volume and heart rate to arrive at a specific target output.

## Abstract (highlights)

- **What is the central question of this study?**
  Cardiac output supposedly matches an increased peripheral demand in healthy humans. Conversely, the mismatch of supply and demand is a hallmark of cardiovascular diseases, such as hypertension and heart failure. Here, we partly separated the local blood flow supply and demand to examine whether cardiac output responds specifically to the peripheral demand in healthy humans.
- **What is the main finding and its importance?**
  The cardiac output response to local heat stress and submaximal exercise does not appear to be entirely specific to the peripheral thermal and energetic requirements. Interpretation of clinical presentations might need to be altered, because even the healthy heart does not supply a specific cardiac output during heat stress and exercise.

## 1 INTRODUCTION

Matching of an increased peripheral demand with a proportionate increase in blood supply is a fundamentally accepted concept in cardiovascular regulation (Bada et al., 2012; Hargreaves & Spriet, 2020b, 2020a; Nyberg & Jones, 2022). Accordingly, a proportionate increase in cardiac output and blood flow to local tissues, such as the skin or the skeletal musculature, is often observed during physiological challenges (Mortensen et al., 2005; Watanabe et al., 2024). The convective delivery of flow by the cardiovascular system necessary to match the peripheral demand is achieved owing to feedback control via several reflexes (baroreflex, muscle metabo‐ and chemoreflexes, also known as the exercise pressor reflex; Alam & Smirk, 1937; Boushel, 2010; Raven, 2008; Raven et al., 2019; Rowell & O'Leary, 1990; Wan et al., 2020). All these reflexes are thought to activate the sympathetic pathways and thereby have the potential to influence stroke volume (SV) and heart rate (HR) via inotropic and chronotropic modulation. Thus, it can be assumed that cardiac output is typically involved when peripheral stimuli trigger a physiological response that requires more blood flow for thermoregulation, increased energetic requirements or blood pressure maintenance. Yet, the precision with which cardiac output contributes to such a peripheral–central matching of the demands of fundamental stimuli is still not fully known. The concept of ‘matching’ relies on the assumption that the healthy heart must either sense the precise peripheral demand and adjust its output accordingly (via control of SV and HR) or that there is at least such natural interdependence between changes in SV and HR that they somehow adjust to produce a specific cardiac output. Failing that, the cardiac output could, alternatively, be controlled to regulate blood pressure, which means that SV and HR would be adjusted not in relationship to peripheral demand but to maintain mean arterial pressure (MAP). Interestingly, some previous studies have suggested that both can be achieved simultaneously, for example when manipulating HR during exercise but observing a similar cardiac output and MAP (Bada et al., 2012; Munch et al., 2014). Alternative evidence suggests that cardiac output is not controlled specifically to the prevailing demand (Stöhr, 2022). Hence, there might, in fact, not be direct biological coordination between SV and HR to arrive at a specific volume in accordance with peripheral demand. Consequently, at present, it remains unclear whether cardiac output is specific to the peripheral demand.

Two common conditions that necessitate an enhanced peripheral perfusion are heat stress and exercise. In both, cardiac output increases in line with the blood flow that is needed peripherally. In the case of heat stress, the increase in cardiac output is almost entirely attributable to the increased perfusion of the skin for the dissipation of heat (Travers et al., 2022). In contrast, during exercise the increased peripheral demand stems from the augmented energetic requirements associated with muscular contraction (Hargreaves & Spriet, 2020b, 2020a; Travers et al., 2022). To date, no study has purposefully investigated whether the typical cardiac output response to either condition is specific to the peripheral demand. Separating the enhanced perfusion of local areas from their demand allows one to determine whether cardiac responses are specific. Therefore, this study sought to investigate the responses to local heat stress and submaximal exercise in normal conditions and during mild lower‐limb blood flow restriction (BFR). Application of BFR will simultaneously alter both local perfusion and venous return, thereby forcing the system to control SV and HR to ensure the constant delivery of a specific cardiac output according to the prevailing peripheral thermal and energetic demands. We hypothesized that cardiac output would fail to match the peripheral demand during heat stress (van Mil et al., 2016), but that, in comparison, cardiac output would be controlled according to demand during exercise (Munch et al., 2014; Silva et al., 2019).

## 2 MATERIALS AND METHODS

### 2.1 Ethical approval and study population

Following approval by the Leibniz University Hannover Ethics Committee (approved protocol ID: LUH/295), a total of 15 individuals volunteered to partake in the study (women/men *n* = 6/9). Owing to illness in the second trial (*n* = 1, female), poor ultrasound windows (*n* = 2, female) and high blood pressure (*n* = 1, male) the final cohort that completed both trials successfully was composed of 11 healthy males and females (women/men = 3/8; age 26 ± 2 years; height 176 ± 9 cm; body mass 73 ± 11 kg), who completed a health questionnaire and provided verbal and written informed consent to take part in the study. Volunteers were not enrolled if they were currently taking any medication other than contraception‐related drugs or if they had a history of cardiovascular disease. Volunteers were recruited from university staff and the student population. The study conforms to the standards set by the latest revision of the *Declaration of Helsinki* (World Medical Association, 2024).

### 2.2 Experimental protocol

Each participant attended the laboratory on a single occasion for each protocol [two visits per participant in total; one for double‐leg heating (DLH) and one for exercise (EX)]. Details of the protocols and measurements are provided below. Data collection was performed during the following three time points: (1) at baseline (Pre^DLH^ or Pre^EX^); (2) immediately after either 30 min of DLH (Normal^DLH^) or after 5 min of supine cycling at 60 W (Normal^EX^); and (3) after 3 min of blood flow restriction (BFR^DLH^ or BFR^EX^) caused by upper thigh cuffing at the prevailing MAP of each individual while DLH or exercise continued. Data collection included echocardiography, whole‐body metabolism via breath‐by‐breath analysis, continuous blood pressure assessment, skin temperatures and capillary lactate measurements. In order to ensure that the physiological reactions to the respective intervention had stabilized, data were collected only when a plateau of skin temperatures and oxygen consumption (${\dot{V}}_{O_{2}}$) was achieved. Skin temperature assessments, blood pressure measurements (LabChart, ADInstruments Pty Ltd, Bella Vista, NSW, Australia), breath‐by‐breath metabolic data (COSMED, COSMED Deutschland GmbH, Werneck, Germany) and cardiac ultrasound images (Vivid E95; GE Healthcare, Trondheim, Norway) were recorded according to current guidelines and as described previously (Stöhr et al., 2011, 2012, 2014). Lactate concentration was determined according to current guidelines during the last 30 s of each experimental condition. Continuous data were averaged over 1 min during the penultimate minute of the experimental condition. Echocardiography was obtained at rest and during the last 90 s of each experimental condition.

### 2.3 Specific procedures

Upon arrival at the laboratory, anthropometric data (date of birth, height and body mass) were recorded, and blood pressure was measured using a commercially available oscillometric cuff‐based blood pressure device (Mobil‐O‐Graph; IEM, Aachen, Germany). Subsequently, two blood pressure cuffs were attached to the upper thighs of the participant, as proximal as possible on both legs. Six skin temperature sensors (ML309 and MLT422/A, ADInstruments, Oxford, UK) were then placed on the participant's skin. The sensors were attached distal to the thigh cuffs, specifically to the distal part of the vastus lateralis muscle, and to the lower legs, centred on the caput mediale of the gastrocnemius muscle. Additionally, two control sensors were attached: one on the lower abdomen, centred on the rectus abdominis muscle, and one on the right forearm, centred on the brachioradialis muscle. Core temperature was not measured in this study, because a previous experiment indicated that this should be unaffected by this level of local skin heating (van Mil et al., 2016). A continuous blood pressure monitoring device (NIBP; ADInstruments) was attached to the intermediate phalange of the middle finger of the right hand and calibrated according to the manufacturer's guidelines. Respiratory gases were collected by fitting a suitable face mask that was connected to volume and O_2_/CO_2_ sensors and the data captured on a breath‐by‐breath basis (Quark CPET, COSMED Deutschland GmbH), previously calibrated in accordance with the manufacturer's instructions. An ECG was also recorded for each participant using the Vivid E95 ultrasound device (GE Healthcare). Three electrodes were attached for this purpose: one on the left clavicle, one on the right clavicle and one on the lower right thorax of the subject. Blood samples were obtained from the right earlobe during each experimental condition and analysed for lactate concentration (C‐Line, EKF Diagnostics, Barleben, Germany). After all measurement devices were attached, the participants lay down on a supine cycle ergometer (Ergoselect 1200P; Ergoline GmbH, Bitz, Deutschland). For baseline measurements and during the DLH trial, both legs were suspended such that they were resting in a straight angle to the hip to ensure a neutral position and prevent strain on the core and leg muscles. During the exercise trial, following baseline measurements both feet were strapped into the cycle ergometer pedals. For each experimental condition, the supine cycle ergometer was tilted 45° left laterally to maximize the quality of cardiac ultrasound images.

### 2.4 Leg heating protocol

In the leg heating protocol, silicone tube‐lined trousers (Med‐Eng, Ottawa, ON, Canada) were used (Gagnon et al., 2016; Pearson et al., 2014). These were connected to a water circulator (Julabo F12 Corio CD‐200F1; Labortechnik GmbH, Seelbach, Germany) set at a pumping flow rate of 15 L min^−1^ 0.35 bar^−1^‐. Before beginning the heating protocol, the water circulating through the trousers was preheated to 50°C and was maintained at this temperature throughout the experiment. Following the baseline measurements (Pre^DLH^), the preheated trousers were rapidly pulled over the legs and additionally wrapped with a thermal foil to provide enhanced insulation and thus a more rapid heating process. Subsequently, the participants were positioned in a neutral supine position until the temperature of the legs reached a plateau. This was usually the case after ∼30 min. The participants were then tilted 45° left laterally again, and data collection was repeated (Normal^DLH^).

### 2.5 Cycling protocol

For the cycling protocol of the study, participants’ feet were strapped into a supine cycle ergometer (Ergoselect 1200P) after the baseline measurements (Pre^EX^). Subsequently, the study participants engaged in a 5 min cycling exercise at an intensity of 60 W in a neutral supine position, with the objective of reaching a plateau in oxygen uptake (${\dot{V}}_{O_{2}}$) and carbon dioxide production (${\dot{V}}_{CO_{2}}$). The intensity of 60 W was chosen because in a previous pilot study with six participants, a slightly lower intensity (50 W) yielded interesting results regarding the cardiovascular response. The pilot study also showed that participants were still working submaximally at 50 W, regarding respiratory exchange ratio (RER) and HR response. Therefore, we decided that an increase to 60 W would amplify the cardiovascular response while still being submaximal for the participants. This was confirmed for all participants by the RER and HR responses during exercise without BFR. The participants were then tilted 45° left laterally again, and data collection was repeated (Normal^EX^). We defined submaximal cycling as aerobic exercise at an intensity where participants were mostly oxidizing substrates such as glucose or fatty acids in order to generate ATP (RER < 1.0, HR < 70% of expected maximal HR based on 220 minus age).

### 2.6 BFR protocol

After the cycling or leg heating data collection phase, the blood pressure cuffs on the thighs were rapidly inflated to the MAP of the participants. This pressure was chosen because of its effects on peripheral blood flow determined in a previous study (van Mil et al., 2016). In addition, extensive pilot work for this project revealed that cuffing at lower pressures [we tested the approach: (MAP − DP)/2 + DP] did not have the desired effect on venous return and, most importantly for our purposes, end‐diastolic volume and SV. Equally, we did not want to perform a full occlusion because this would probably have caused a smaller circuit and entrapment of a certain blood volume in the legs. Our approach is also supported by a recent study evaluating cardiovascular physiology (Mladen et al., 2025). After 3 min of adjustment, all measurements were repeated. Finally, for both heat stress and exercise conditions, the central blood pressure and haemodynamics of the participant were assessed in a supine position with a Mobil‐O‐Graph (IEM). Participants reported no experiences of pain or discomfort attributable to BFR in both trials.

### 2.7 Data collection and analysis

#### 2.7.1 Echocardiography

As previously stated, the data were collected in accordance with the current guidelines. Three to five cardiac cycles were recorded at normal, unforced end–expiration with an M5S probe on a commercially available ultrasound system (Vivid E95; GE Healthcare) and analysed with the manufacturer's software (EchoPAC software only version 204 revision 02; GE Healthcare). One sonographer was responsible for collecting and analysing the images of all participants. Left ventricular volumes, HR and cardiac output were determined from triplane images and analysed using manual tracing of the endocardial border in diastole and systole.

#### 2.7.2 Continuous brachial blood pressure and central haemodynamics

Blood pressure was measured using calibrated continuous photoplethysmography (NIBP; ADInstruments) and recorded for later analysis of mean arterial, systolic and diastolic blood pressures. Central blood pressure and haemodynamic measurements were obtained using an oscillometric cuff‐based blood pressure device (Mobil‐O‐Graph; IEM).

#### 2.7.3 Whole‐body metabolism

To assess the respiratory gas exchange during exercise, breath‐by‐breath analysis of respiratory volume and contents was performed using the Quark CPET system (COSMED Deutschland GmbH). The primary variables measured were ${\dot{V}}_{O_{2}}$ and ${\dot{V}}_{CO_{2}}$. Each participant was fitted with an appropriately sized face mask, which was securely connected to the device to capture and analyse respiratory gases. Throughout both heat stress and exercise, ${\dot{V}}_{O_{2}}$ and ${\dot{V}}_{CO_{2}}$ were measured continuously and recorded breath by breath. Data were analysed to determine key respiratory and metabolic parameters, including the RER and the minute ventilation (VE).

#### 2.7.4 Lactate

During both trials, blood samples were obtained from the right earlobe to determine lactate concentrations at the three experimental time points. Blood sampling was conducted with minimal discomfort using a sterile lancet. The collected blood samples were promptly analysed using the C‐Line device (EKF Diagnostics). The analyses were carried out in accordance with the manufacturer's protocol to ensure consistency and reliability. All measurements were logged into a secure database immediately after analysis for subsequent statistical evaluation. Quality control procedures included the routine calibration of the C‐Line device before each set of measurements, in addition to periodic validation against known standard lactate solutions.

### 2.8 Statistical analyses

For continuously recorded data, the arithmetic mean of the penultimate minute of each experimental time point was used for the statistical analysis. Descriptive data are reported as means ± SD. Outcomes of statistical comparisons are reported as means ± 95% confidence intervals (CI). Baseline differences were assessed using Student's paired *t*‐test. Differences in the rate of temperature rise between Normal^DLH^ and BFR^DLH^ were assessed using Student's paired *t*‐test. Two‐way repeated‐measures ANOVA was used to determine the effect of BFR and any interaction effect between heat stress and exercise. For central blood pressure data, mixed‐effects model analysis was used for *n* = 11 participants during the DLH trial and *n* = 8 participants for the exercise trial. All data were analysed using GraphPad Prism 10 for Windows (v.10.3.1; GraphPad Software, San Diego, CA, USA).

## 3 RESULTS

**FIGURE 1:**
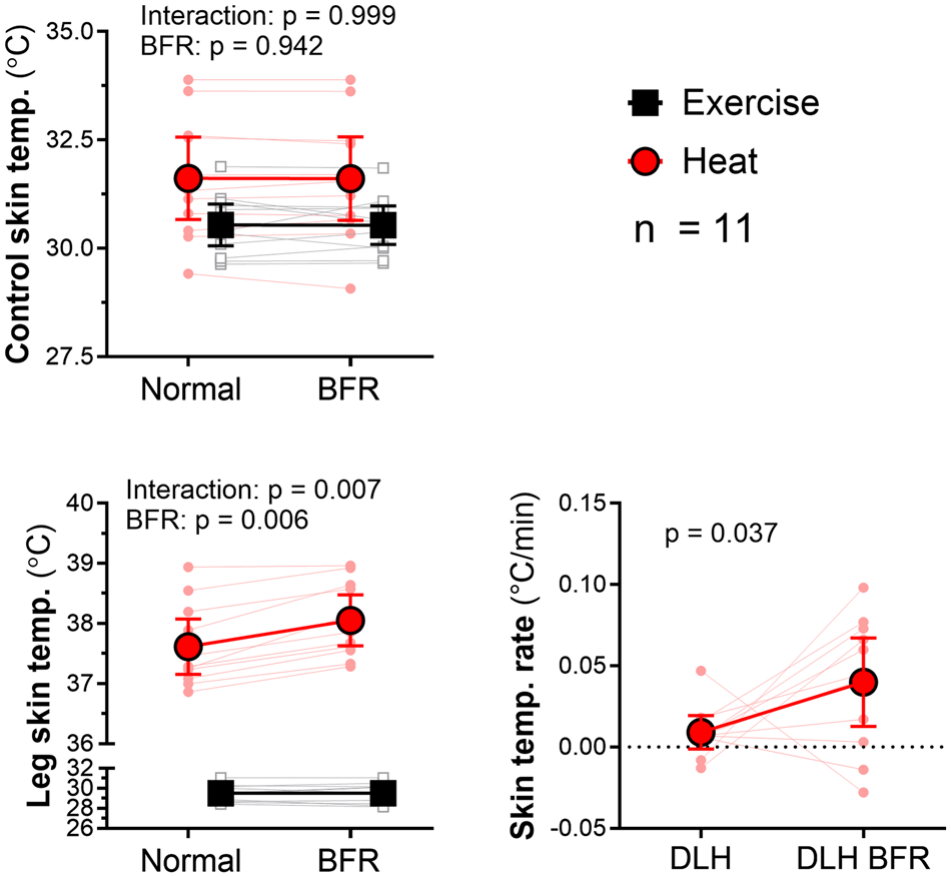
Skin temperatures. In control areas (forearm and abdomen), BFR did not alter skin temperatures. However, leg skin temperatures increased further when BFR was applied during heating, evidenced by a significantly greater rate of temperature rise in the BFR condition. Results of two‐way repeated‐measures ANOVA (control skin temperature and leg skin temperature; *n* = 11) and Student's paired *t*‐test (skin temperature rate; *n* = 11). Abbreviations: BFR, blood flow restriction; DLH, double‐leg heating.

**FIGURE 2:**
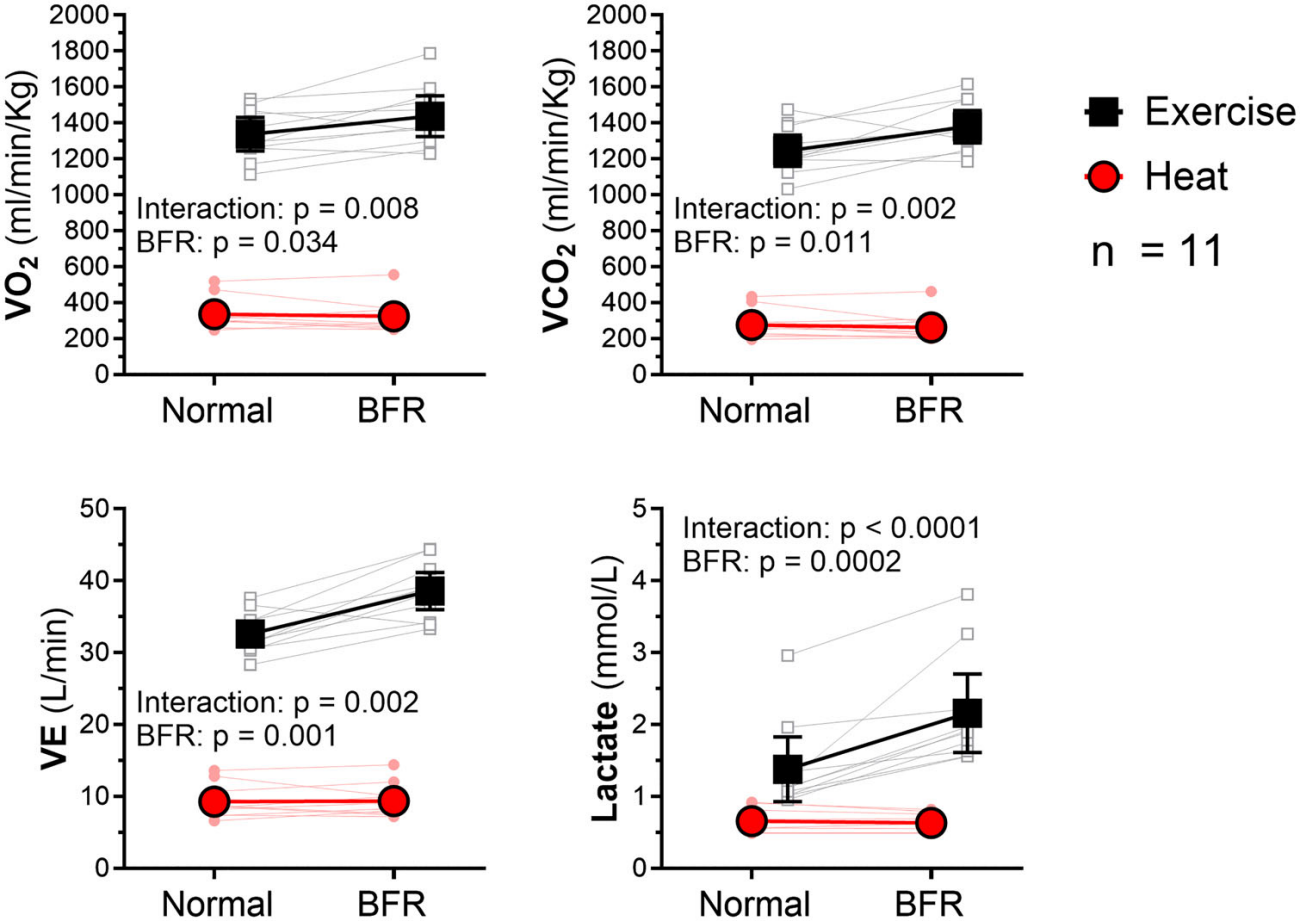
Metabolism during heat stress and exercise with BFR. The ${\dot{V}}_{O_{2}}$, ${\dot{V}}_{CO_{2}}$, VE and lactate increased significantly more during EX^BFR^ compared with DLH^BFR^. Results of two‐way repeated‐measures ANOVA (*n* = 11). Abbreviations: BFR, blood flow restriction; DLH, double‐leg heating; EX, exercise; ${\dot{V}}_{CO_{2}}$, carbon dioxide production; VE, ventilatory equivalent; ${\dot{V}}_{O_{2}}$, oxygen consumption.

**FIGURE 3:**
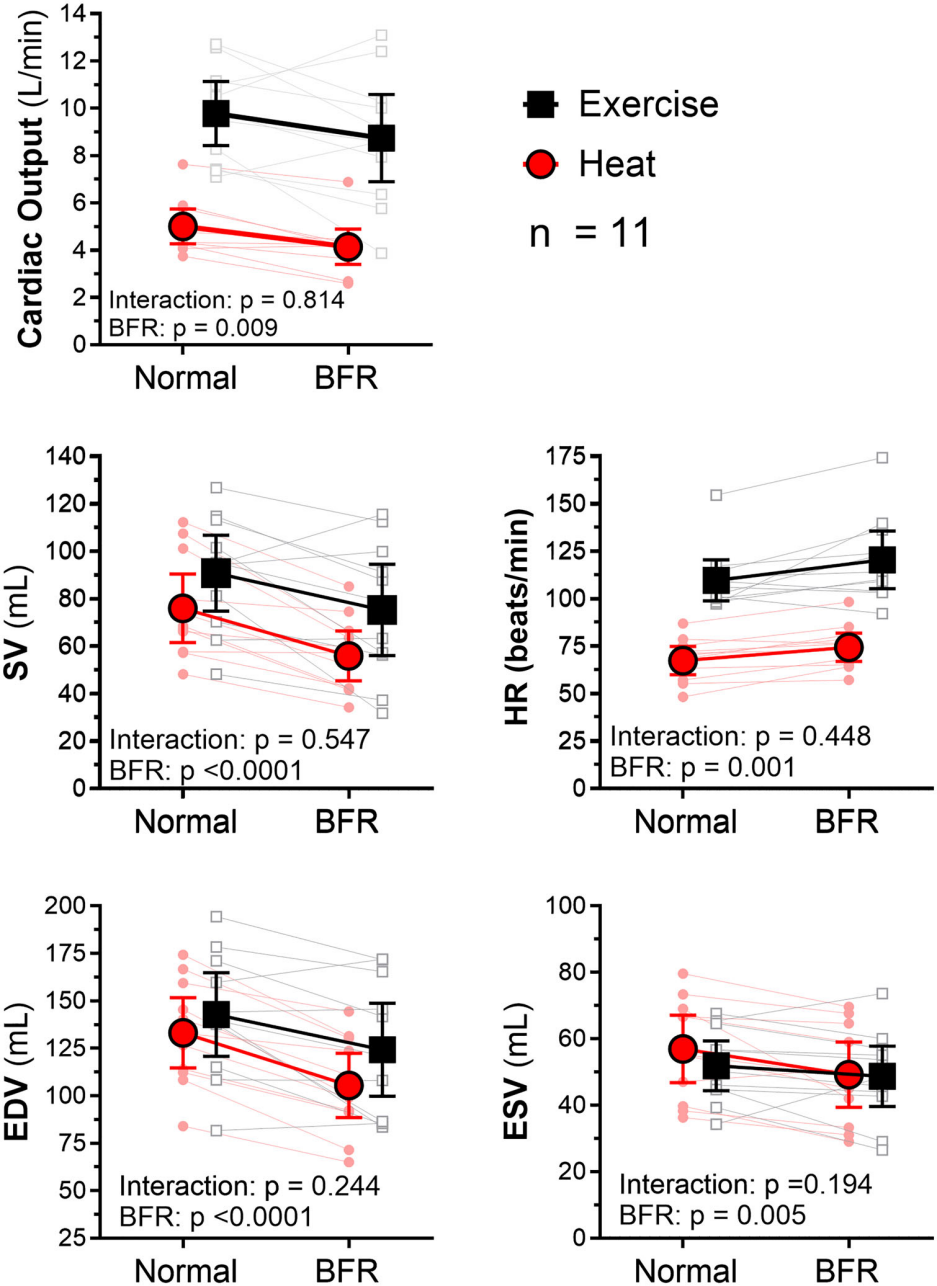
Cardiac output during heat stress and exercise. BFR caused a significant reduction in EDV, ESV, SV and cardiac output, whereas HR increased. These responses were similar during heat stress and exercise. Results of two‐way repeated‐measures ANOVA (*n* = 11). Abbreviations: BFR, blood flow restriction; DLH, double‐leg heating; EDV, end‐diastolic volume; ESV, end‐systolic volume; HR, heart rate; SV, stroke volume.

**FIGURE 4:**
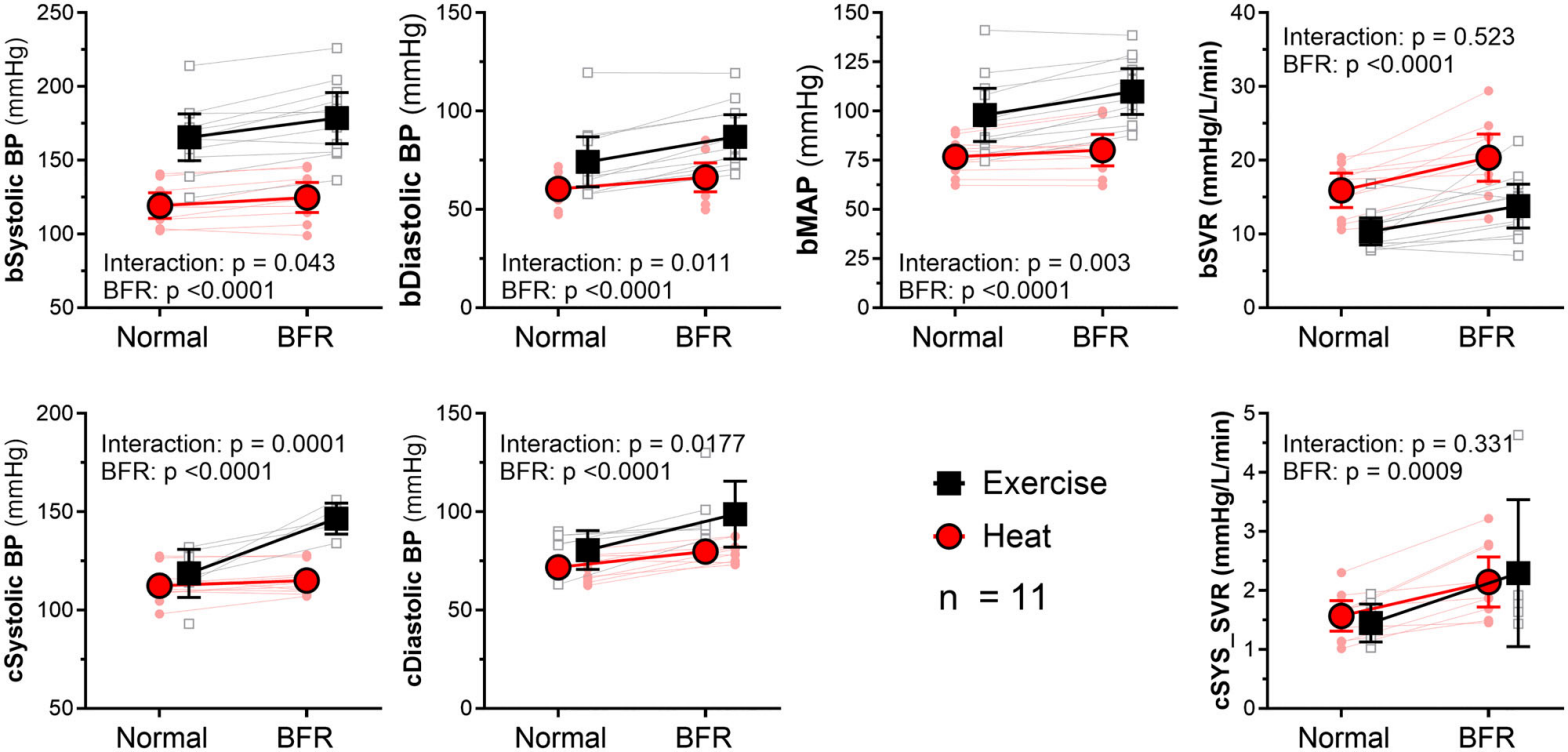
Blood pressure during heat stress and exercise with BFR. Despite a lower cardiac output during BFR, blood pressure increased significantly. Furthermore, the increase in BP was significantly greater during exercise compared with heat stress. Interestingly, central systolic resistance (central systolic BP in relationship to SV) appeared to be similar between DLH and exercise, and increased only marginally (0.5–1.0 mmHg L^−1^ min^−1^), albeit with a much greater variability during exercise. Results of two‐way repeated‐measures ANOVA (*n* = 11) and mixed‐effects model analysis used for *n* = 11 participants during the DLH trial and *n* = 8 participants for the EX trial. Abbreviations: b, brachial; BFR, blood flow restriction; c, central; DLH, double‐leg heating; EX, exercise; SV, stroke volume.

**FIGURE 5:**
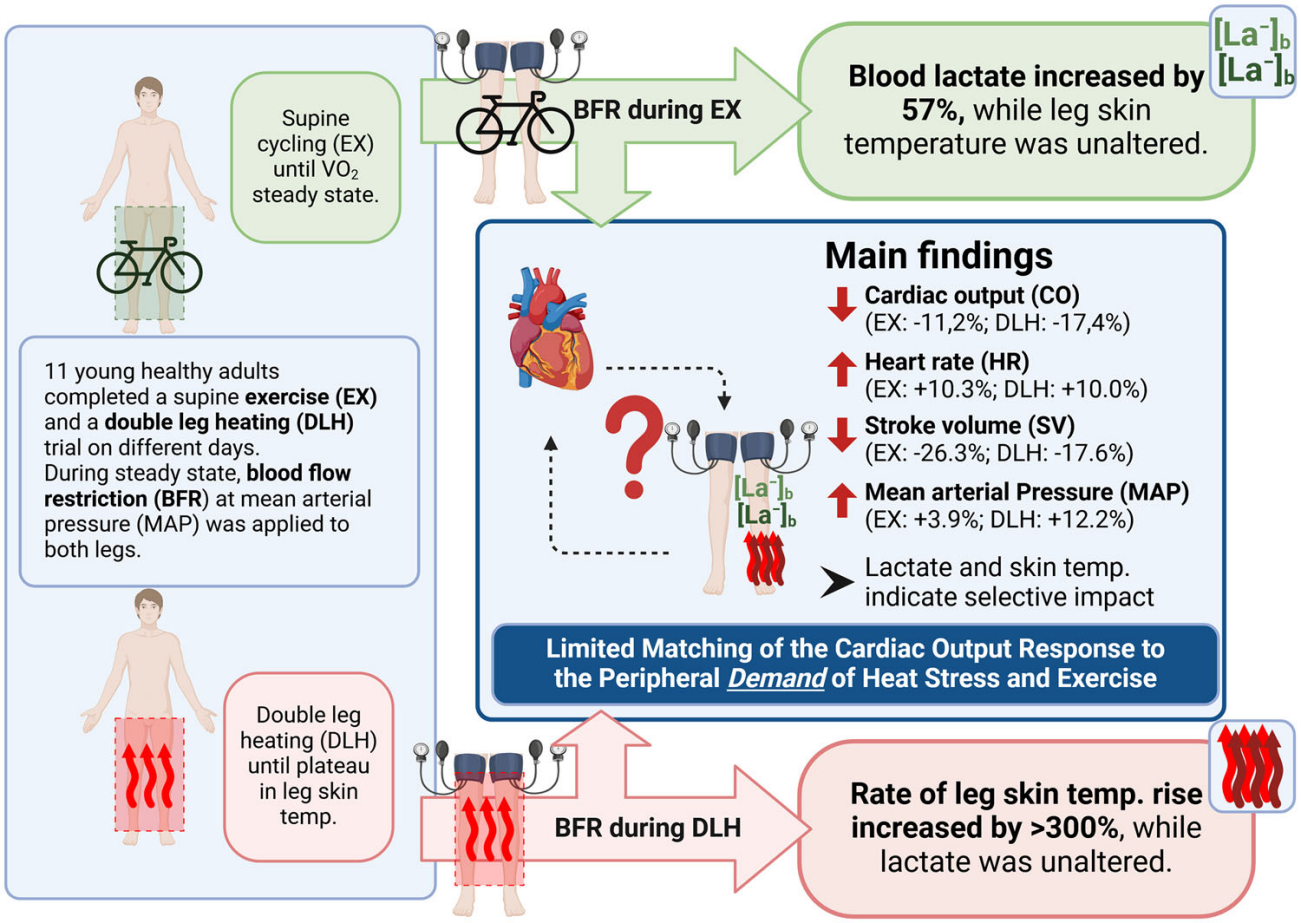
Schematic illustration summarising the study design and the main findings. Eleven healthy participants underwent double‐leg heat stress (DLH) and submaximal exercise (EX) in normal conditions and with blood flow restriction (BFR) at mean arterial pressure (MAP). The normal cardiac output (CO) during heat stress and exercise significantly decreased with BFR, affecting thermoregulation and metabolism. This indicates limited matching of the cardiac output response to the peripheral demand of heat stress and exercise. Created in BioRender. Lampkemeyer, M. (2025) https://BioRender.com/n18i623

There were no baseline differences between the heat stress and exercise trials, except for central systolic blood pressure (*p* = 0.031; Table 1). All experimental data are presented in Table 2 and Figures 1, 2, 3, 4, and we concentrate here, for the purpose of clarity, on the main outcome parameters. A concise summary of the study design and the main findings is illustrated in Figure 5.

**TABLE 1:** Baseline comparisons.

Parameter	Pre^DLH^			Pre^EX^			95% CI
Age, years	26	±	2				
Height, cm	176	±	8				
Body mass, kg	73	±	11				
bSBP, mmHg	126	±	16	123	±	16	−23 to 16
bDBP, mmHg	61	±	10	57	±	16	−19 to 10
bMAP, mmHg	78	±	1	74	±	15	−19 to 11
bSVR	17.1	±	3.5	18	±	5.1	−1.6 to 3.4
cSBP	113	±	9	118	±	9	1 to 10^*^
cDBP	73	±	3	75	±	6	−2 to 5
cSYS SVR	1.5	±	0.3	1.6	±	0.4	−0.13 to 0.37
HR, beats min^−1^	62	±	7	58	±	7	−19 to 10
SV, mL	76	±	16	76	±	20	−23 to 22
CO, L min^−1^	4.73	±	1.03	4.32	±	0.9	−2.12 to 1.31
EDV, mL	137	±	25	140	±	29	−29 to 33
ESV, mL	61	±	15	64	±	15	−12 to 17
${\dot{V}}_{O_{2}}$, mL min^−1^	334	±	75	353	±	85	−98 to 137
${\dot{V}}_{CO_{2}}$, mL min^−1^	282	±	65	304	±	79	−80 to 125
VE	9.2	±	1.4	9.6	±	2.1	−0.6 to 1.5
Lactate, mmol L^−1^	0.78	±	0.23	0.78	±	0.23	−0.48 to 0.49

*Note*: Values are reported as the means ± SD; 95% CI are reported for Student's paired *t*‐test of baseline measurements.

Abbreviations: bDBP, brachial diastolic blood pressure; bMAP, brachial mean arterial pressure; bSBP, brachial systolic blood pressure; bSVR, brachial systemic vascular resistance; cDBP, central diastolic blood pressure; CI, confidence interval; CO, cardiac output; cSBP, central systolic blood pressure; cSYS SVR, central systolic systemic vascular resistance; DLH, double‐leg heating; EDV, end‐diastolic volume; ESV, end‐systolic volume; EX, exercise; HR, heart rate; SV, stroke volume; ${\dot{V}}_{CO_{2}}$, carbon dioxide production; VE, ventilatory equivalent; ${\dot{V}}_{O_{2}}$, oxygen consumption.

* *p* = 0.031.

**TABLE 2:** Intervention comparisons.

Parameter	Normal	BFR	95% CI BFR	95% CI IA	*p*‐Value BFR	*p*‐Value IA
bSBP^DLH^, mmHg	119 ± 13	125 ± 15	6–13	0–14	<0.0001	0.043
bSBP^EX^, mmHg	166 ± 24	179 ± 26				
bDBP^DLH^, mmHg	60 ± 8	66 ± 11	7–12	2–12	<0.0001	0.011
bDBP^EX^, mmHg	74 ± 19	87 ± 17				
bMAP^DLH^, mmHg	77 ± 12	80 ± 12	5–10	3–14	<0.0001	0.003
bMAP^EX^, mmHg	98 ± 20	110 ± 17				
bSVR^DLH^, mmHg L min^−1^	15.9 ± 3.5	20.3 ± 2.7	−5.5 to 2.3	−2.2 to 4.2	<0.0001	0.523
bSVR^EX^, mmHg L min^−1^	10.3 ± 4.8	13.8 ± 4.4				
cSBP^DLH^, mmHg	112 ± 9	119 ± 13	9–18	14–33	<0.0001	0.0001
cSBP^EX^, mmHg	115 ± 7	147 ± 8				
cDBP^DLH^, mmHg	72 ± 6	81 ± 11	7–15	−3 to 14	<0.0001	0.177
cDBP^EX^, mmHg	80 ± 5	99 ± 16				
cSYS SVR^DLH^, mmHg L min^−1^	1.6 ± 0.4	1.5 ± 0.3	0.4–1.2	−0.42 to 1.2	0.0009	0.331
cSYS SVR^EX^, mmHg L min^−1^	2.1 ± 0.6	2.3 ± 1.2				
HR^DLH^, beats min^−1^	68 ± 11	75 ± 11	4–14	−6 to 14	0.001	0.448
HR^EX^, beats min^−1^	110 ± 16	121 ± 23				
SV^DLH^, mL	76 ± 22	56 ± 16	−25 to −10	−11 to 20	<0.0001	0.547
SV^EX^, mL	91 ± 24	75 ± 29				
CO^DLH^, L min^−1^	5.01 ± 1.11	4.13 ± 1.12	−1.7 to −0.26	−1.56 to 1.24	0.009	0.814
CO^EX^, L min^−1^	9.77 ± 2.03	8.73 ± 2.75				
EDV^DLH^, mL	133 ± 28	105 ± 25	−31 to −15	−7 to 25	<0.0001	0.244
EDV^EX^, mL	142 ± 33	124 ± 37				
ESV^DLH^, mL	57 ± 15	49 ± 15	−9 to −2	−3 to 12	0.005	0.194
ESV^EX^, mL	52 ± 11	49 ± 14				
VE^DLH^, L min^−1^	9.3 ± 2.3	9.4 ± 2.2	1.8–4.2	3.4–8.3	<0.0001	<0.0001
VE^EX^, L min^−1^	32.6 ± 2.8	38.6 ± 3.9				
${\dot{V}}_{O_{2}}$ ^DLH^, mL min^−1^	336 ± 84	323 ± 88	4–83	33–192	0.034	0.008
${\dot{V}}_{O_{2}}$ ^EX^, mL min^−1^	1338 ± 139	1438 ± 168				
${\dot{V}}_{CO_{2}}$ ^DLH^, mL min^−1^	277 ± 77	262 ± 77	15–105	59–238	0.011	0.002
${\dot{V}}_{CO_{2}}$ ^EX^, mL min^−1^	1245 ± 129	1379 ± 135				
RER^DLH^	0.82 ± 0.05	0.81 ± 0.07	−0.01 to 0.03	0–0.08	0.336	0.062
RER^EX^	0.93 ± 0.04	0.96 ± 0.04				
Lactate^DLH^, mmol L^−1^	0.66 ± 0.17	0.63 ± 0.13	0.21–0.54	0.47–1.14	0.0002	<0.0001
Lactate^EX^, mmol L^−1^	1.38 ± 0.63	2.16 ± 0.77				

*Note*: Results of two‐way repeated‐measures ANOVA are reported as rounded means ± SD. Statistical comparisons for measurements of central blood pressure were performed as a mixed‐effects model analysis owing to missing values and a reduced sample size compared with the rest of the manuscript.

Abbreviations: bDBP, brachial diastolic blood pressure; BFR, blood flow restriction; bMAP, brachial mean arterial pressure; bSBP, brachial systolic blood pressure; bSVR, brachial systemic vascular resistance; cDBP, central diastolic blood pressure; CO, cardiac output; cSBP, central systolic blood pressure; cSYS SVR, central systolic systemic vascular resistance; DLH, double‐leg heating; EDV, end‐diastolic volume; ESV, end‐systolic volume; EX, exercise; HR, heart rate; IA, interaction; SV, stroke volume; ${\dot{V}}_{CO_{2}}$, carbon dioxide production; VE, ventilatory equivalent; ${\dot{V}}_{O_{2}}$, oxygen consumption.

In addition to the anticipated increase in skin temperatures with DLH and no change in skin temperatures with EX, the rate of increase in skin temperature reached a plateau prior to the collection of data at DLH, but subsequently increased significantly in response to DLH^BFR^ (+344%, *p* = 0.037; Figure 1, 5).

The ${\dot{V}}_{O_{2}}$ and ${\dot{V}}_{CO_{2}}$ did not change with BFR during DLH, and there was a mild increase in ${\dot{V}}_{O_{2}}$ and ${\dot{V}}_{CO_{2}}$ during EX (Table 2; Figure 2). Lactate concentration was significantly influenced by BFR (*p* = 0.0002; 95% CI: 0.21, 0.54) and increased significantly more during EX^BFR^ (interaction *p* < 0.0001; 95% CI: 0.47, 1.14; Figure 2, 5).

With the application of BFR, cardiac output reduced similarly during DLH and EX (BFR *p* = 0.009; 95% CI: −1.7, −0.26; interaction *p* = 0.814; 95% CI: −1.56, −1.24) because of significant reductions in SV, while HR increased mildly (BFR *p* < 0.0001; 95% CI: −25, −10; and BFR *p* = 0.001; 95% CI: 4, 14, respectively), but no interaction effect between DLH and EX trials was found (interaction *p* = 0.547; 95% CI: −11, 20; interaction *p* = 0.448; 95% CI: −6, 14, respectively; Figure 3, 5).

During both DLH and EX, systolic and diastolic blood pressure increased mildly, albeit slightly more with BFR during EX (Table 2; Figure 4, 5).

## 4 DISCUSSION

Based upon the results from our experiments, we can accept our hypothesis only in part. On the one hand, we found evidence that agrees with the idea that the cardiac output response to heat stress is unspecific. On the other hand, we found that the cardiac output response to submaximal exercise was also unspecific. To place the present findings into context, we initially describe in more detail what we mean by an ‘unspecific’ cardiac output.

### 4.1 ‘Unspecific’ cardiac output – the importance of a cardiovascular reserve

Obviously, any cardiac output is always specific to a situation, because it occurs in certain circumstances. However, it has been assumed that for a given stimulus and associated peripheral response (e.g., via an increase in peripheral demand) a precise and adequate cardiac response will be triggered (Bada et al., 2012). During local heat stress without changes in core temperature, the component of blood that is most relevant for local temperature regulation is the plasma volume. In contrast, during exercise the component of blood that is most relevant is the amount of O_2_ to meet the local metabolic demand of the contracting musculature. Since cardiac output cannot separate into the blood components such as plasma volume or O_2_ molecules, this already indicates that any delivery might have side‐effects on other aspects of the circulation, thus increasing the chances of an unspecific response. Moreover, to test the hypothesis of whether the cardiac output matches the peripheral demand and can thus be considered truly specific to that, three things need to be ensured. First, it is essential that the response of an individual to a condition be measured as a reference point. Second, the cardiovascular system needs to have the flexibility (or reserve) to up‐ or downregulate. In other words, the prevailing demand needs to be elevated from the resting condition such that it can be downregulated within the normal physiological range, but it must not be elevated maximally such that there also remains the possibility to upregulate. Third, and perhaps most importantly, the demand needs to be separated from the perfusion, to determine whether it has an influence on its own. In contrast to previous studies, which achieved the first two aspects (Bada et al., 2012; Gonzalez‐Alonso et al., 2008; Munch et al., 2014), our experimental design also achieved the third aspect by choosing mild heat stress (other studies provoked higher skin temperatures; Pearson et al., 2011, 2014; Watanabe et al., 2024) and submaximal exercise, as evidenced by a RER of < 1.0, in addition to an alteration of the perfusion of the limbs while the original stimulus (heat or exercise) was kept the same. This is crucial in relation to our view of an ‘unspecific’ cardiac output, because the strict peripheral demand for components of blood to the skin for heat dissipation or to the muscles owing to augmented energetic requirements of submaximal exercise was the same, but two significantly different cardiac outputs were observed. In short, when the stimulus and peripheral demand are the same but different cardiac outputs are registered, we conclude that the cardiac output must be ‘unspecific’ to the demand associated with the original stimulus. The present results indicate that there was no reduction in demand for blood flow during the BFR trials, because temperature increased during DLH^BFR^, and ${\dot{V}}_{O_{2}}$ increased during EX^BFR^.

### 4.2 Evaluation of the present results

The responses during heat stress with BFR agree with one further study performed with local arm heating (van Mil et al., 2016). Conversely, the responses to exercise and BFR are not in accordance with previous studies, which showed a maintained or increased cardiac output (Karabulut & Garcia, 2017). However, previous studies used suprasystolic cuff inflation pressures to induce BFR. It is likely that such an approach does not only separate the perfusion from the demand (as intended in our experiment) but also alters the demand. In our study, this was probably not the case, because the same BFR had differential effects during the DLH^BFR^ and EX^BFR^ trials, and those effects were specific to the main physiological requirements. This was evidenced by a maintained ${\dot{V}}_{O_{2}}$ during DLH^BFR^ and a maintained skin temperature during EX^BFR^, while ${\dot{V}}_{O_{2}}$ and lactate increased during EX^BFR^, and leg skin temperatures increased during DLH^BFR^. Consequently, the present study shows that the effect of the BFR was specific to the condition that caused the increased demand for blood flow, whilst not affecting normal baseline perfusion. In the future, it would be interesting to combine leg heat stress with cycling exercise to determine whether the specificity of responses might cause a biological competition between tissues or whether the effects are simply additive.

Considering that the demand itself was mostly maintained, the observation of an increase in the rise of the leg skin temperature during DLH^BFR^ suggests that the reduced cardiac output was too low to raise blood pressure sufficiently to perfuse the skin at the same rate as during DLH without BFR. Given that the blood flow during heat stress is almost exclusively required for its plasma volume for heat transfer, this part of the study shows that peripheral requirements for fluid do not seem to be sensed precisely and that the output from the heart does not seem to be controlled specifically according to it. This might have important implications for conditions in which there is a problem with the fluid content and distribution to different end‐organs. Taken together, we interpret the cardiac output response to DLH^BFR^ as suboptimal and suggest that future studies should investigate more specifically the local perfusion in the skin and the role of central and peripheral blood pressure regulation during similar conditions.

In the present study, the responses during heat stress were corroborated during submaximal exercise. The application of BFR resulted in a reduction in SV in both trials, which is likely to be attributable to a decline in venous return (Ozaki et al., 2010), as supported by the reduction in left ventricular end‐diastolic volume in this study. The impaired SV was neither fully compensated for by a reduction in end‐systolic volume (which might have been ‘blocked’ because of higher total peripheral resistance; see later) nor by a further increase in HR, both factors that could have been altered by increased sympathetic activation. Accordingly, increases in HR and MAP during DLH with BFR might have resulted from elevated sympathetic activity as a compensatory attempt to maintain perfusion in conditions of reduced venous return. DLH might also have induced vasodilatation to facilitate heat dissipation, which would have been associated with enhanced peripheral cutaneous perfusion. We think that BFR exacerbated the distal vasodilatation whilst reducing venous outflow, which could have increased cardiac sympathetic drive to sustain cardiac output and MAP. Reflexes, such as baroreflex modulation and increased cutaneous afferent signalling, might also have contributed to the observed responses, although the involvement of the baroreceptors remains to be studied. Specifically, the current knowledge on baroreceptor ‘resetting’ might be applied, in part, to the BFR condition. However, it is not clear whether such a resetting would explain the lack of matching between local peripheral flow demand and the delivery from the heart via cardiac output. At present, therefore, we interpret the decline in cardiac output as suboptimal (like the decline during heat stress), also because of the concomitant increase in lactate concentrations whilst ${\dot{V}}_{O_{2}}$ did not reduce. This increase in lactate at a low absolute level coupled with an increase in RER most probably reflects a slight shift in the substrate utilization towards a greater reliance on glucose. This might be caused by mild local tissue hypoxia in the thighs induced by BFR, which results in a reduced blood flow and therefore less oxygen delivery to the stressed muscles. This could lead to alterations in metabolism, with a decrease in the proportion of fat oxidation and an increase in carbohydrate utilization. The switch might ultimately happen owing to the lower oxygen requirements for the generation of ATP through oxidative phosphorylation of glucose (Hargreaves & Spriet, 2020a, 2020b). The increased concentration of metabolites as a result of venous occlusion and the mechanical changes by BFR might elicit an even greater response of the exercise pressor reflex, which leads to increased muscle fibre recruitment via group III and IV afferent pathways (Herring et al., 2024; Loenneke et al., 2011). Increased activation of these pathways might also explain, in part, the mildly increased VE and CO_2_ production, because they might account for ∼50% of hyperpnoea during exercise (Iannetta et al., 2024). Presumably, muscle glycogen was used for ATP production, because it is the main source of carbohydrates during intense exercise(Hargreaves & Spriet, 2020a, 2020b). Likewise, it might be possible that feedforward mechanisms contributed to the increased HR. Less likely, although not improbable, is an increased contribution of anaerobic metabolism that could also have been caused by mild ischaemia induced by BFR. Equally, we cannot completely exclude the possible role of different muscle activation during BFR in our study. Owing to the counter‐pressure on the contracting musculature caused by BFR, it is possible that a greater activation of muscles was required to perform the same exercise task at the fixed absolute intensity. Equally, it is possible that a change in the activation of the skeletal musculature with BFR caused a shift towards an increased recruitment of type II muscle fibres. Furthermore, the increased lactate might be attributable to a shift in muscle fibre type activation. Previous studies have reported increased EMG amplitudes, which might indicate an increased recruitment of type II muscle fibres and possibly explain the increased ${\dot{V}}_{O_{2}}$ and ${\dot{V}}_{CO_{2}}$ levels observed in the present and previous studies, because they require more oxygen to produce the same amount of energy and thus produce more CO_2_ (Mendonca et al., 2015). The fact that lactate decreased with BFR during heat stress indicates that this response was specific to exercise. Nonetheless, in both case, the lower cardiac output has to be interpreted as a negative, suboptimal response that was inferior to the response without BFR, despite reserves in SV and HR.

### 4.3 Implications: cardiac (and possibly arterial) movement occurs for unknown reasons

The main findings are of importance for our immediate understanding of the role of the heart during periods of increased physiological demands, such as heat stress and exercise. However, the findings have an even greater relevance when the implications are considered. If the consequence of the present data is that the heart does not ‘know’ how much blood the periphery needs and its output is not precise, what is the role of the heart? And what does this mean for our interpretation of heart ‘disease’ (the main ones, hypertension and heart failure, which are typically characterized by a reduction in the heart's output)? There is the possibility that the myocardial contraction and relaxation are more specific and precise, beyond the typical factors of preload, afterload and contractility. For example, left ventricular twist has been reported to be similar in athletes with larger hearts and larger SVs (Cooke et al., 2018; Weiner et al., 2015). Likewise, pregnant women have a similar left ventricular twist to non‐pregnant women (Meah et al., 2019). These data indicate that myocardial deformation can occur similarly but generate different outputs. Accordingly, the factors that govern myocardial deformation might be more specifically controlled than those regulating myocardial output. Understanding these factors would be important to understand disease processes and generate new therapeutic strategies.

### 4.4 Limitations

Ideally, we would have liked to measure skeletal muscle blood flow to quantify the amount of flow that perfused the limbs during the experimental conditions. Because blood flow in the common femoral artery is extremely difficult to measure during supine cycling exercise, we relied on previous publications and a body of literature that has already examined blood flow accordingly. In future studies, we would like to add this measurement and perhaps also include other peripheral parameters, such as the oxygenation of the skeletal muscle and the activation of muscle fibres.

In the literature, systemic blood pressure is often measured in one location (brachial artery) to represent the whole circulation. It has to be acknowledged that this might not necessarily be the case in circumstances such as our experimental approach, and local pressure measurements additionally to systemic pressure assessments would present an advancement to our understanding of the components of the cardiovascular system.

### 4.5 Translational perspective

A close interdependence between HR and SV is expected in the regulation of blood pressure and supply–demand matching within the healthy human cardiovascular system. Conversely, the hallmark of many cardiovascular diseases are haemodynamic load alterations and supply–demand mismatch (e.g., hypertension and heart failure). The present study shows that even the healthy heart does not supply a specific cardiac output during heat stress and exercise, with consequences for absolute blood pressure. Accordingly, the interpretation of clinical presentations might need to be altered, offering new opportunities for alternative research avenues.

## 5 CONCLUSION

The cardiac output response to local heat stress and submaximal exercise does not appear to be specific to the peripheral thermal and energetic demand. This finding supports the theory that even the healthy heart does not coordinate SV and HR to arrive at a specific target output or blood pressure. Therefore, cardiovascular regulation (and cardiac contraction and relaxation in particular) might take place for reasons other than the ‘matching’ between convective flow delivery, local demand and, therefore, blood pressure.

## AUTHOR CONTRIBUTIONS

All experiments were performed in the COR‐HELIX laboratory at the Institute of Sports Science of the Leibniz University Hannover. Moritz Lampkemeyer, Jonas Kell, Veit Börß, Tobias Claussen, Craig G. Crandall and Eric J. Stöhr conceptualized and designed the study. Data acquisition was performed by Moritz Lampkemeyer, Jonas Kell, Veit Börß, Tobias Claussen, Fabian Spahiu, Michelle Ottlik, Lars C. Helbig and Eric J. Stöhr. Data analysis and interpretation was performed by Moritz Lampkemeyer, Jonas Kell and Eric J. Stöhr. Moritz Lampkemeyer and Eric J. Stöhr drafted the manuscript. All authors critically revised the manuscript, approved its final version and agree to be accountable for all aspects of the work in ensuring that questions related to the accuracy or integrity of any part of the work are appropriately investigated and resolved. All persons designated as authors qualify for authorship, and all those who qualify for authorship are listed.

## CONFLICT OF INTEREST

The authors declare that they do not have any conflict of interest to report in relationship to this manuscript.

## Data Availability

The datasets generated during and/or analysed during the present study are available from the corresponding author on reasonable request.
